# Cancer-Targeted Controlled Delivery of Chemotherapeutic Anthracycline Derivatives Using Apoferritin Nanocage Carriers

**DOI:** 10.3390/ijms22031362

**Published:** 2021-01-29

**Authors:** Katarzyna Kurzątkowska, Manuel A. Pazos, Jason I. Herschkowitz, Maria Hepel

**Affiliations:** 1 Department of Chemistry, State University of New York at Potsdam, Potsdam, NY 13676, USA; 2 Department of Biosensors, Institute of Animal Reproduction and Food Research, Polish Academy of Sciences, Tuwima 10 Str., 10-748 Olsztyn, Poland; 3 Department of Biomedical Sciences, Cancer Research Center, University at Albany, State University of New York, Rensselaer, NY 12222, USA; rdevaux@albany.edu (M.A.P.II); jherschkowitz@albany.edu (J.I.H.)

**Keywords:** apoferritin nanocarriers, apoferritin model calculations, drug-nanocage interactions, anthraquinone drugs’ hydrophobic interactions, controlled drug delivery, idarubicin, ferritin receptor targeting, folate receptor targeting

## Abstract

The interactions of chemotherapeutic drugs with nanocage protein apoferritin (APO) are the key features in the effective encapsulation and release of highly toxic drugs in APO-based controlled drug delivery systems. The encapsulation enables mitigating the drugs’ side effects, collateral damage to healthy cells, and adverse immune reactions. Herein, the interactions of anthracycline drugs with APO were studied to assess the effect of drug lipophilicity on their encapsulation excess *n* and in vitro activity. Anthracycline drugs, including doxorubicin (DOX), epirubicin (EPI), daunorubicin (DAU), and idarubicin (IDA), with lipophilicity *P* from 0.8 to 15, were investigated. We have found that in addition to hydrogen-bonded supramolecular ensemble formation with *n* = 24, there are two other competing contributions that enable increasing *n* under strong polar interactions (APO(DOX)) or under strong hydrophobic interactions (APO(IDA) of the highest efficacy). The encapsulation/release processes were investigated using UV-Vis, fluorescence, circular dichroism, and FTIR spectroscopies. The in vitro cytotoxicity/growth inhibition tests and flow cytometry corroborate high apoptotic activity of APO(drugs) against targeted MDA-MB-231 adenocarcinoma and HeLa cells, and low activity against healthy MCF10A cells, demonstrating targeting ability of nanodrugs. A model for molecular interactions between anthracyclines and APO nanocarriers was developed, and the relationships derived compared with experimental results.

## 1. Introduction

Recent advances in cancer treatment, achieved through the development of precision guided surgical techniques, novel drugs, and modern technologies for cancer detection and monitoring [1,2,3,4], have resulted in considerable deceleration of the disease progression and spread. However, the number of new cancer cases is still growing. Therefore, a great effort is still being paid to solve key challenges in cancer therapy by focusing on early detection [5], nanotechnology-assisted theranostics [4], overcoming multi-drug resistance (MDR) [6,7,8,9,10], control of proliferation and metastasis, and activation of immunodefenses [11]. The advantages of using chemotherapeutic agents with high cytotoxicity and an ability to kill cancer cells are often offset by harmful side effects, MDR, and DNA damage to healthy cells [12], which may preclude prolonged treatment due to organ failure or systemic collapse. There is now a growing body of evidence that these antagonistic effects can largely be prevented by developing controlled drug delivery (CDD) systems [1,13,14,15] based on biocompatible drug nanocarriers with the targeting ability to act upon cancer cells. In this work, we have addressed some of these issues by investigating the capability of a biogenic nanocage carrier, apoferritin (APO), to encapsulate anthracycline (ANTR) chemotherapeutic drugs for safe, controlled delivery directly to the cancer cells, thus mitigating most of the adverse effects encountered in a systemic drug delivery in classical chemotherapy [2,3,4,5,12,16].

The interactions of ANTR derivatives with APO nanocages are the key features for drug loading and transporting through the biological medium. However, the lack of systematic investigations of these interactions prevents successful development of APO-based drug delivery systems. In this work, we propose a novel comprehensive physicochemical model of these interactions showing non-trivial dependence of the drug loading on various types of interactions, including hydrogen bonding, polar interactions, hydrophobic interactions, and supramolecular complex formation, in addition to the usually considered simple entrapment of drugs in solution-filled APO nanocages. The mathematical modelling presented should be useful to gain better understanding of not only the drug binding but also the drug releasing processes, induced by pH change and APO opening in the intracellular environment of a cancer tissue.

In vitro testing of the activity of APO-encapsulated anthracyclines, including doxorubicin (DOX), epirubicin (EPI), daunorubicin (DAU), and idarubicin (IDA), widely differing in their lipophilicities, was performed using flow cytometry and cytotoxicity/growth inhibition methods for HeLa cervical cancer and MDA-MB-231 adenocarcinoma breast cancer cells, as well as non-tumorigenic MCF10A epithelial cells.

The nanocarrier-based CDDs can provide significant benefits to anticancer therapy, including targeted drug delivery (TDD) [13,14,15], protection of drugs against deactivation by the bioenvironment [2,17], increased drug solubility, extended durability in the bloodstream [18], and improved uptake by cells [2]. The nanocarriers may also carry adjuvants and immunoreaction suppressors to aid in therapy. The important capability of TDD, associated with incorporation of targeting ligands on the nanocarrier surface, is based on the recognition and binding to the receptors overexpressed in cancer cell membranes [19], thus enabling targeted endocytosis of the nanocarriers [16,20,21].

The CDD system may also serve as an efficient vehicle to overcome MDR [6,7,10], which is challenging due to the involvement of alterations in several signaling pathways. MDR is acquired by tumors as a protection against reactive oxygen species (ROS), the main weapon of the organism to fight cancer [22,23,24,25], by overexpressing anti-apoptotic proteins and glutathione (GSH) [26,27], able to neutralize highly active radicals of ROS. Due to MDR, an increased dose of chemotherapeutic agents is necessary to overcome drug resistance, inevitably leading to increased collateral damage to healthy cells. Therefore, the nanocarrier-based TDDs become the forefront technology in cancer treatment since the nanocarriers can deliver higher drug doses directly to the tumor tissue without damaging healthy cells [4,12,16].

Different kinds of nanoparticle (NP) nanocarriers have been tested in preclinical studies, but only a few of them have been so far clinically approved because of concerns with toxicity, immunogenicity, or sequestration by the reticuloendothelial system [28]. On the other hand, naturally occurring NPs offer low toxicity and the ability to evade immune detection. One such a biological NP proposed for CDD is ferritin [29,30], an iron storage protein with perfect biocompatibility and biosafety [31]. Ferritin is a multimeric protein [32] consisting of subunits self-assembled into a nanocage structure of apoferritin (APO), with an outer diameter of 12 nm and an inner diameter of 8 nm [32,33]. The inside cavity of an APO can encapsulate molecular cargos (e.g., drugs) and be reversibly disassembled/assembled into/from subunits depending on the environmental pH [32,33]. The APO nanocages are highly stable and are resistant to both high temperatures (up to 70 °C) and wide pH range (from 2.0 to 10.0) [28,34]. The cancer growth is supported by acidosis developed in its microenvironment and ATP expression to fulfill its energy needs for rapid proliferation [35]. Targeting of tumor cells is achieved through the abilities of the transferrin receptor 1 (TfR1) and the Scavenger Receptor Class A Member 5 (SCARA5), overexpressed in cancer cells [19,36] to recognize APO nanocages. Hence, the APO nanostructure is a promising vehicle for targeted CDD. It has already been shown that several drugs, such as cisplatin, streptomycin, doxorubicin, and daunomycin, can be encapsulated in APO [29,30,37,38,39,40,41,42,43,44,45,46]. Recently, epirubicin-carrying APO nanocarriers have been applied to study MCF-7 breast cancer cells survivability upon nanodrug treatment [47]. Drug encapsulation efficiency of 7.7% has been achieved. The APO nanocarriers have also been proposed for the delivery of drug photosensitizers [48], and curcumin-based protectors against hepatocellular damage [49].

Anthracyclines, investigated in this work, are antibiotics that inhibit DNA and RNA synthesis by intercalating into the nucleic acid duplex [50,51], damaging the DNA and cell membranes [52]. Anthracyclines are used to treat leukemias and solid tumors, including lung and breast cancers, gynecological cancers, and sarcomas. A major limitation for the use of anthracyclines is cardiotoxicity [50,52,53]. To reduce the side effects, various biomolecular nanocarriers have been considered [30,43,44,45,46,54,55].

**Figure 1:**
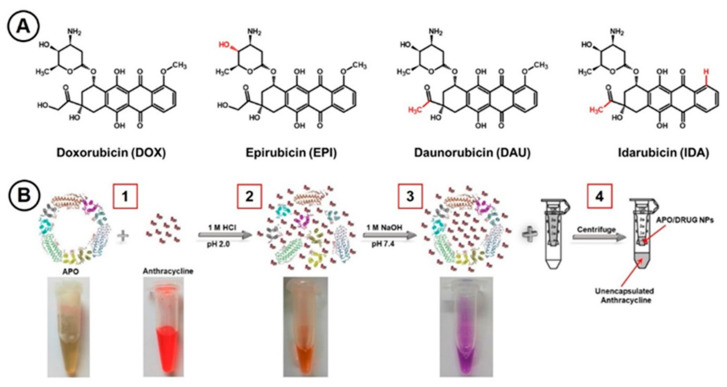
(**A**) Structures of anticancer drugs of the anthracycline group. (**B**) Schematic illustration of anthracycline encapsulation in an apoferritin nanocage forming an APO (drug) nanocarrier. The steps 1 to 4 are described in the text.

In this work, we have investigated the immobilization processes of anthracycline drugs in APO nanocages, followed by monitoring the drug release rate using fluorescence spectroscopy. The investigations involved four clinically relevant anthracyclines (Figure 1A), widely differing in their lipophilicity, ranging from *P* = 0.8 to 15.0. We hypothesize that, owing to the richness of binding sites on the inside surface of the protein cavity, APO can bind, via supramolecular interactions, drug molecules with different lipophilicities and enable efficient encapsulation of the drugs for targeted delivery. While doxorubicin has been extensively studied before [29,30,38,39,40,41,42,43,44,45,46], due to its clinical use, a comprehensive study of the nanodrugs based on APO-encapsulated DOX, EPI, DAU, and IDA, to our knowledge, has not been published. To enhance the APO nanocarriers’ targeting ability, we have employed amide binding chemistry to attach folic acid targeting ligands directly to the external surface of APO nanocarriers. This procedure eliminates also the use of toxic thiols for ligand binding to the Au film on APO, recently proposed [56]. The overexpression of folate receptor (FR) in many cancer cells, in addition to TrfR1 and SCARA5, enhances the APO targeting ability in nanodrug anticancer therapy [16,57]. The in vitro testing of APO nanodrugs’ activity was performed using flow cytometry and cytotoxicity/growth inhibition methods for standard HeLa cells, MDA-MB-231 breast cancer adenocarcinoma cells, and non-tumorigenic MCF10A epithelial cells.

## 2. Results

### 2.1. Anthracycline Encapsulation in Apoferritin Nanocages

To load anthracycline drugs into APO nanocages, pH-mediated reversible disassembly/re-assembly processes in solutions of free drugs have been carried out. The subunits of an apoferritin shell have been disassembled by applying an acidic environment (pH 2.0) and then re-assembled back to form the nanocage structure with encapsulated drugs by returning to physiological conditions (pH 7.4) [45]. Schematic illustration of the sequence of processes for encapsulation of ANTR molecules in an APO nanocage is presented in Figure 1B. In these experiments, APO (1 mg/mL) was mixed with a drug (1) at different molar ratios, as described below. The solution pH was then lowered to 2.0 to disassemble the APO structure (2).

The mixture was shaken for 15 min to create a homogeneous solution. In the next step, the pH was returned to neutral by adding NaOH and the mixture was incubated for 2 h to enable re-assembly of APO nanocages with encapsulated ANTR molecules (3), thus creating the APO(drug) NPs. The APO re-assembly was corroborated by the color change of the mixture from red to purple. The prepared NPs were centrifuged using Amicon-Ultra-0.5 mL 30K to remove free drug molecules (4). The APO(drug) nanocarriers were then rinsed with Milli-Q water and centrifuged five times at relative centrifugal force RCF = 14,000× *g* for 10 min.

To determine the optimum molar concentration ratio of drug:APO for the encapsulation solution, the tests were performed using doxorubicin as the model anticancer drug, using the ratios of DOX:APO from 50:1 to 200:1. For the ratios from 100:1 to 200:1, the encapsulation molar ratio *n* = [drug]/[APO] was found to be close to 31.5, indicating that a saturation of nanocages was achieved. Therefore, for further studies, the molar ratio of drug:APO = 100:1 was selected for all drugs under study to minimize the amount of the drugs used. The encapsulation molar ratios for different ANTR derivatives in APO nanocages are presented in Table 1.

The highest encapsulation ratio was observed for IDA (33.6) which also shows the highest lipophilicity of 15.0, mainly due to the absence of group -O-CH_3_ at carbon C4. For the rest of the anthracyclines investigated, the encapsulation ratios are decreasing from 31.5 to 29.3 with increasing drug lipophilicity from 0.8 to 3.5. For this group of anthracyclines (DOX, EPI, DAU), the observed encapsulation ratios follow the decrease in dipolar interactions between the drugs and APO with increasing drug lipophilicity.

**Figure 2:**
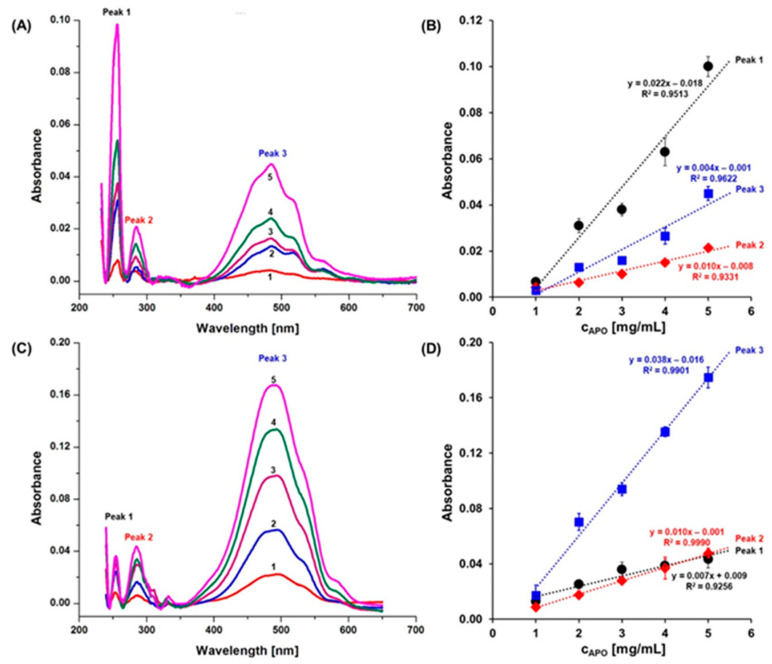
The UV-Vis spectra for solutions of: (**A**) APO(IDA) and (**C**) APO(DOX) nanocarriers, for increasing concentrations of APO nanocages [mg/mL]: (1) 1; (2) 2, (3) 3, (4) 4, (5) 5. (**B**,**D**) Dependences of the peak absorbance intensities vs. APO concentration for: (**B**) APO(IDA) and (**D**) APO(DOX) nanocarriers for peak P1 at 255 nm, peak P2 at 280 nm, and peak P3 at 485 nm. Samples were diluted 1:10 with Milli-Q water. Data are expressed as mean ± SD (*n* = 5) and the values of correlation coefficient *R* for linear fitting are printed at the curves.

As seen in Figure 2, the UV-Vis spectra for APO(IDA) and APO(DOX) nanocages show three characteristic peaks at: 255 nm (P1), 280 nm (P2), and 485 nm (P3). Apoferritin itself exhibits an absorption band at 280 nm (P2, *ε*_280_ = 468,000 cm^−1^ M^−1^), whereas the spectra of anthracyclines show absorption bands P1 and P3. With increasing concentrations of APO, the intensities of all three peaks increase linearly. As seen in the spectra for APO(IDA) nanocarriers in Figure 2, the intensity of peak P1 is higher than that of P2 and their intensity ratio is constant: P1/P2 = 5.5. However, for APO(DOX) nanocarriers, P1 ≤ P2 (the intensity ratio is: P1/P2 = 0.7 to 1.0). The relationship between the intensities of peak P3 and P2 for APO(IDA) and APO(DOX) is similar: P3 > P2 but their intensity ratios differ substantially: P3/P2 = 2.5 for APO(IDA), whereas P3/P2 = 3.8 for APO(DOX).

These data indicate that the interactions of DOX and IDA with APO nanocages differ significantly. The linear dependence of the intensity of P2 on APO concentration *C*_APO_ confirms that no aggregation of APO(drug) nanocarriers occurs.

### 2.2. Circular Dichroism Spectroscopy

**Figure 3:**
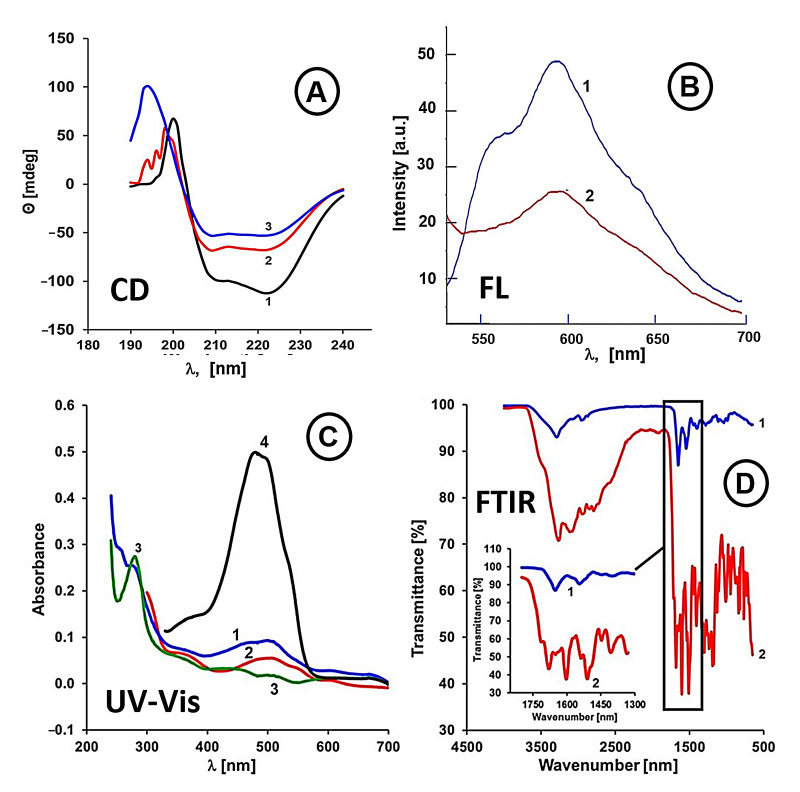
(**A**) Circular dichroism spectra for a 1 mg/mL solution of a wild-type APO with and without an encapsulated drug: (1) bare APO; (2) APO(IDA) nanocarrier; and (3) APO(DOX) nanocarrier. Spectra were recorded in water at 25 °C in a 0.5 cm quartz cuvette. (**B**) Fluorescence emission spectra (*λ*_ex_ = 480 nm) for nanocarriers: APO(DOX) (blue line, **1**), APO(DOX)@FA (red line, **2**). (**C**) UV-Vis spectra for: APO(DOX) (blue line, **1**), APO(DOX)@FA (red line, **2**), APO (green line, **3**), and DOX (black line, **4**). (**D**) FTIR spectra of APO(DOX) (blue line, **1**) and APO(DOX)@FA (red line, **2**) nanocages.2.3. Attachment of Folate Targeting Ligands to APO Nanocages.

To obtain further information concerning the interactions of anthracycline drugs with APO nanoshells, we have applied circular dichroism (CD) spectroscopy which enables evaluation of conformation changes in the APO protein during the encapsulation of ANTRs. Two anthracycline drugs with the largest difference in their lipophilicity were taken for comparison. The CD spectra were recorded in the far-UV spectral region from 190 to 240 nm (Figure 3A). The spectrum for wild-type APO exhibits typical features of an α-helical protein with two characteristic minima around 210 and 220 nm, and a maximum at 200 nm.

The percentage of α-helix for APO alone was predicted to be 92.6%. After the encapsulation of anthracyclines, the spectra of APO(IDA) and APO(DOX) revealed deviations in both minima and maximum from their original pattern, indicating that the α-helix content in the APO structure has changed. For APO(IDA), the percentage of α-helix decreased and was predicted to be 89.9%, whereas for the APO(DOX), it slightly increased to reach 93.2%. Thus, IDA with its stronger hydrophobic properties disrupts the α-helical APO structure, while DOX, with more pronounced dipolar interactions, stabilizes the APO α-helix.

Modifications of the outer surface of APO nanocages with a gold film and functional ligands have recently been proposed [56]. Herein, to improve the effectiveness of APO(drug) nanocages in TDD, we modified the APO surface with a folic acid (FA) targeting ligand via EDC/NHS activation of carboxylic groups of FA, followed by the attachment of FA to –NH_2_ groups on the APO surface. The attached folate moiety binds to a folate receptor (FR) exposed in cancer cell membranes. The best-known isoform, FRα, is a glycosyl phosphatidylinositol-anchored glycoprotein. It is overexpressed by cancer cells. Upon binding the folate-conjugated nanocarriers, it can internalize them through the receptor-mediated endocytosis [57].

To confirm the attachment of FA to the APO(drug) surface, detailed measurements using FTIR, UV-Vis, and fluorescence spectroscopies, with an APO(DOX) as the model nanocarrier have been performed. As seen in Figure 3B, the fluorescence spectrum of APO(DOX)@FA shows a decrease of ca. 50% of the peak intensity from that observed for APO(DOX) emission at *λ*_em_ = 593 nm. This phenomenon is likely due to the quenching of DOX fluorescence by FA, recently documented by Husseini et al. [58] and Santra et al. [59], thus confirming the presence of a folate moiety on the external surface of an APO(DOX) nanocage.

Figure 3C illustrates the UV-Vis spectra of doxorubicin, bare APO, APO(DOX), and APO(DOX)@FA. Upon DOX encapsulation in the APO nanocage, the DOX absorption band was shifted from 480 nm to 505 nm, while the position of the APO peak at *λ*_max_ = 280 nm remained unchanged (Figure 3C, curve 1). However, binding of FA to the external surface of APO nanocage resulted in a shift of the APO band position from 280 nm to 355 nm (Figure 3C, curve 2).

Further proof of the FA attachment to the nanocarriers was obtained from FTIR spectra for APO(DOX)@FA showing a band for stretching vibration of carbonyl group (–C=O) of the attached FA at 1697 cm^−1^ and a band at 1485 cm^−1^ for stretching vibration of phenyl ring of FA (Figure 3D, curve 2). These bands are absent for APO(DOX) (Figure 3D, curve 1). Based on these data, we can conclude that the attachment of FA on the surface of APO was successful.

The drug release experiments indicate that the FA attachment to APO(DOX) surface has no significant influence on the stability of APO(DOX) nanocarriers.

### 2.3. Cell Death Assay

**Figure 4:**
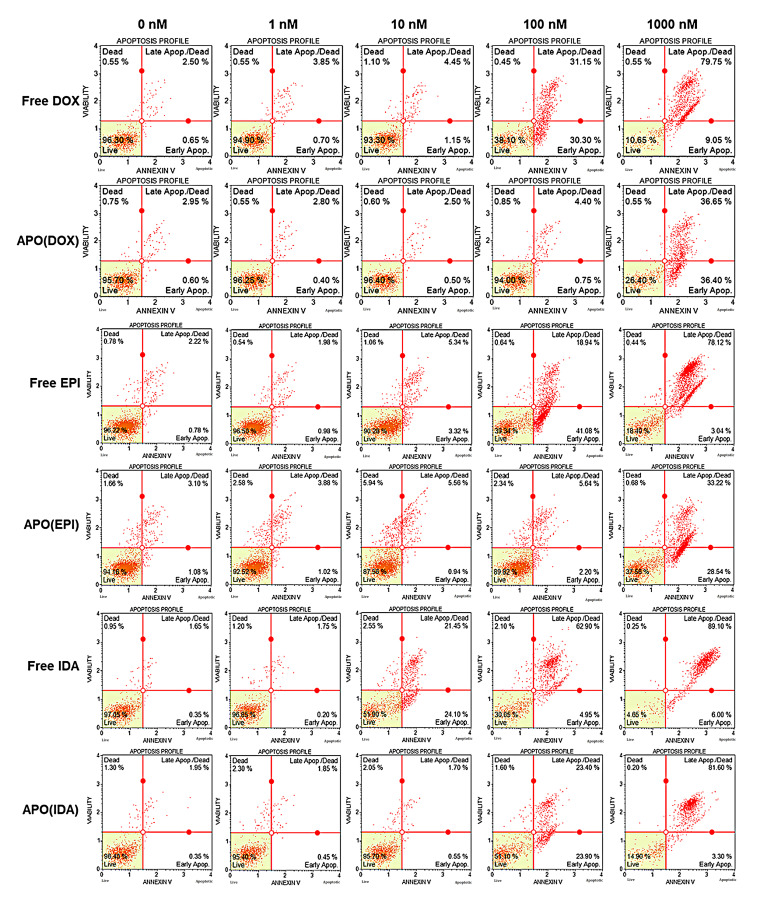
Flow cytometry apoptosis profiles for 7-AA D staining (DNA accessibility index) vs. annexin V staining (membrane damage index) for MDA-MB-231 breast cancer cells treated for 72 h with indicated concentration of anthracycline: (column 1) 0 nM, (column 2) 1 nM, (column 3) 10 nM, (column 4) 100 nM, and (column 5) 1000 nM, using different free anthracyclines (row 1—DOX, row 3—EPI, and row 5—IDA) and APO(drug) nanocarriers with encapsulated anthracyclines (row 2—APO(DOX), row 4—APO(EPI), and row 6—APO(IDA)).

**Figure 5:**
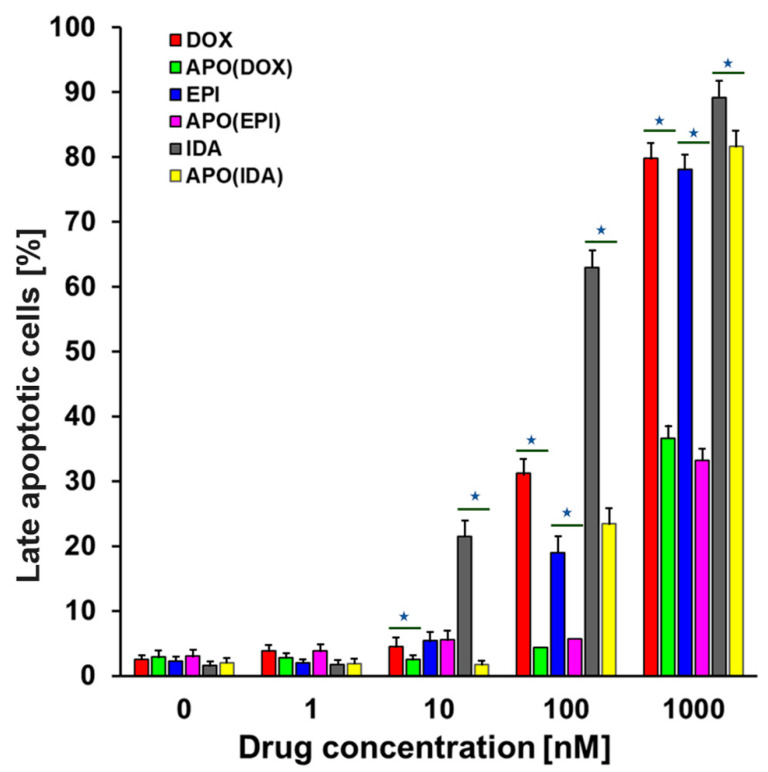
Dependence of the fraction of late apoptotic cells, found after 72 h treatment of MDA-MB-231 breast cancer cells with ANTR drugs, on concentration of drugs, injected as free drugs or in form of APO(drug) nanocages. All data are expressed as mean ± SD (*n* = 5); statistically significant (*P* < 0.05) difference is shown as “⋆”.

Since the primary mechanism of chemotherapeutic drug action is based on the induction of apoptosis, we have investigated how the APO nanocages with encapsulated ANTR drugs fare relative to free drugs. In our experiments, the MDA-MB-231 cancer cells were treated with free ANTRs (DOX, EPI, IDA) at concentrations: 1, 10, 100, and 1000 nM, or different drug-loaded APO nanocages (APO(DOX), APO(EPI), APO(IDA)) containing equivalent amounts of drugs. After 72 h of treatment, the cells were analyzed, and the percentage of apoptotic cells determined by flow cytometry assessment of Annexin-V-stained cells (Figure 4). We have found that APO(DOX) and APO(EPI) nanocarriers are less effective in the induction of apoptosis than the free DOX and EPI drugs, as expected, due to the encapsulation. For instance, the percentage of late apoptotic cells, observed after treatment of MDA MB-231 breast cancer adenocarcinoma cells with APO(DOX) and APO(EPI) at 1000 nM drug concentration, was 36.7 ± 1.5% and 33.2 ± 1.3%, respectively (Figure 5), which is lower than that for free drugs: 79.7 ± 3.2% and 78.1 ± 3.1% for DOX and EPI, respectively (see also: Figure 4, panel row 2 vs. 1, and panel row 4 vs. 3). Significant contribution to the difference between the efficiency of encapsulated and free drugs is due to the slower drug release from the nanocarriers in comparison with the immediate drug availability of the free drugs. The comparison of nanocarriers’ and free drugs’ activity was changed considerably when early apoptotic cells were also included: the total number of apoptotic cells was found to be 73.1 ± 2.9% and 61.8 ± 2.5% for APO(DOX) and APO(EPI), respectively. These numbers are still lower than the respective numbers of apoptotic cells observed after treatment with free drugs: 88.8 ± 3.6% and 81.2 ± 3.2% for DOX and EPI but the difference is much smaller. The observed effect is due to the time-dependency of the drug release from APO(drug) nanocarriers.

Among the anthracyclines examined, free IDA shows the strongest apoptotic action toward MDA-MB-231 cancer cells, with the percentage of late apoptotic cells approaching 89.1 ± 3.6%, and the APO(IDA) nanocarriers also show a high efficiency of 81.6 ± 3.3% (Figure 4, rows 5 and 6). By including early apoptotic cells, these numbers increase further to 95.1 ± 3.8% and 84.9 ± 3.4%, for free IDA and APO(IDA), respectively.

It is important to bear in mind that the encapsulation enables targeting of cancer cells and provides protection against collateral damage to healthy cells. Also, the concentration of drug nanocarriers can be readily increased, if necessary, which is especially advantageous for hydrophobic drugs with limited solubility in body fluids.

### 2.4. Cytotoxicity/Growth Inhibition Assay by Crystal Violet Staining

The cytotoxicity of the APO(drug) nanocarriers was further confirmed using a cytotoxicity/growth inhibition assay. Again, APO(IDA) has shown the highest activity and induced more apoptotic MDA-MB-231 cancer cells than the APO nanocarriers loaded with other anthracyclines. Therefore, for further experiments, we selected APO(IDA) to evaluate the effectiveness of APO nanocarriers in treatment of cancer cells: HeLa and MDA-MB-231, in relation to that of healthy MCF10A cells.

**Figure 6:**
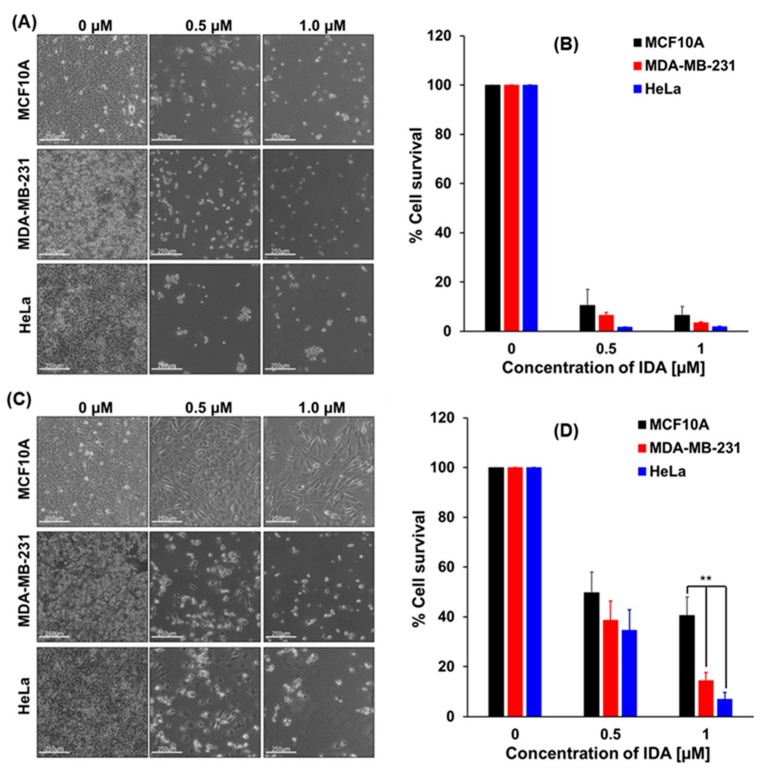
The effects of concentration of free IDA (**A**,**B**) and APO(IDA) (**C**,**D**) on cell growth of MCF10A, MDA-MB-231, and HeLa cells studied by the cytotoxicity/growth inhibition assay with crystal violet staining. (**A**) Representative brightfield images of cells cultured in the presence of free IDA, with IDA concentration, *C*_IDA_ [µM]: 0.0, 0.5 and 1.0. (**B**) Normalized crystal violet signal representing cell survival for experiments in (A). (**C**) Representative brightfield images of cells cultured in the presence of APO(IDA), with equivalent concentration of *C*_IDA_ [µM]: 0.0, 0.5 and 1.0. (**D**) Normalized crystal violet signal representing cell survival for experiments in (**C**). All data are expressed as mean ± SD (*n* = 3); statistically significant differences in cell survival between MCF10A and MDA-MB-231 (*P* = 0.0052) and between MCF10A and HeLa (*P* = 0.0019) is shown as “**”. Scale bars in all images (A,C) are 250 µm.

In Figure 6, the ability of HeLa, MDA-MB-231, and MCF10A cells, treated with free IDA (Figure 6A,B) and APO(IDA) nanocarriers (Figure 6C,D), to form colonies was examined for 72 h. As shown in the images of Figure 6A,C, only a very small number of colonies were formed by all cell lines under study after treatment with free IDA at 1 µM concentration. The lowest cell survival percentage was observed for HeLa cells (2.1 ± 0.3%) and it was also very low for MDA-MB-231 cells (3.2 ± 0.5%) and MCF10A cells (6.0 ± 09%).

The treatment of cancer cells with the 1.0 µM IDA in APO(IDA) nanocages resulted in the death of 86 ± 3% and 93 ± 2% of MDA-MB-231 adenocarcinoma cells and HeLa cervical cancer cells, respectively, compared to 59 ± 8% dead cells observed for MCF10A healthy cells (Figure 6D).

It follows from these data that the encapsulation of idarubicin in apoferritin cavity protects healthy cells against the harmful effects of the drug. Thus, by using APO nanocarriers, the anthracycline chemotherapeutic drugs can be delivered to cancer cells in a targeted manner.

### 2.5. Determination of the Dose–Response Relationships and EC_50_ Values

**Figure 7:**
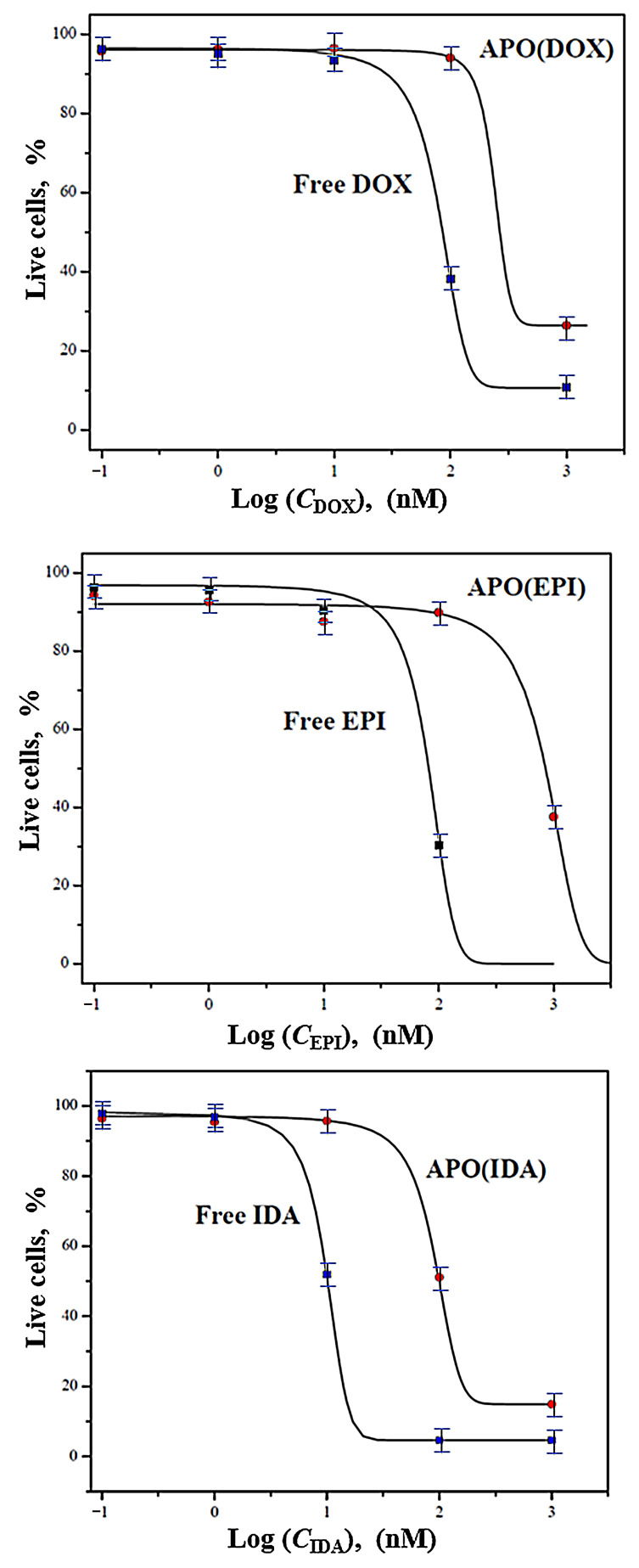
Dose–response curves for free ANTR drugs and APO-encapsulated ANTR nanodrugs, obtained for MDA-MB-231 breast cancer cells.

The dose–response relationships for free ANTR drugs and APO-encapsulated ANTR nanodrugs have been evaluated based on the flow cytometry data obtained for MDA-MB-231 breast cancer cells treated with these drugs and analyzed by the annexin V and 7-ADD staining. The drug dose–response curves are presented in Figure 7. It is seen that for all APO-encapsulated drugs, the dose–response curves are shifted toward higher drug concentrations. This is due to the protective nature of the APO encapsulation and slower drug release. Taking into account the targeting ability of the APO(ANTR) nanodrugs, the drug concentration can be safely increased offering a high dose delivery with reduced collateral damage to healthy cells. The EC_50_ values for free ANTR drugs and APO-encapsulated nanodrugs have been calculated from the dose–response curves and are presented in Table 2.

## 3. Discussion

### 3.1. Mechanisms of Encapsulation of ANTR Drugs

The APO shell nanocarriers offer a unique ability to safely transport highly toxic chemotherapeutic drugs to the targeted tumors while enabling straightforward drug loading and unloading operations. They effectively prevent damage to healthy cells, mitigate the drugs’ side effects, and protect drugs against deactivation by the biological environment. Moreover, the APO nanocarriers do not induce any immunoresponse of the organism. In our experiments, we have demonstrated that chemotherapeutic drugs from the anthracycline group can be encapsulated in APO nanocarriers with high efficiency despite considerable differences in drug lipophilicity ranging from 0.8 to 15. Based on the experiments performed, we have encountered the following mechanisms of encapsulation of these drugs, showing different dependences of drug binding ability on their lipophilicity:

(i) For ANTR drugs with low lipophilicity (*P* = 0.8 to 3.5), the efficiency of overall binding of drugs to the APO nanocarriers decreases with increasing drug lipophilicity, following the trend for polar interactions between the drugs and APO. This trend is consistent with the behavior of DOX, EPI, and DAU.

(ii) For ANTR drugs with high lipophilicity (*P* = 3.5 to 15), where the polar interactions play a reduced role, an opposite trend is observed with the overall drug binding efficiency increasing with the increasing lipophilicity. This trend is characteristic for hydrophobic interactions between the drugs and a matrix and it is consistent with the behavior of DAU and IDA. The interactions of APO with IDA, showing the highest lipophilicity (*P* = 15.0), are the strongest among the drugs examined. This coincides with IDA’s highest encapsulation ratio. Although the hydrogen bonding could play a role here, the detailed analysis of the number of possible hydrogen bonds, the anthracycline derivatives can form with APO, indicates that all the drugs under study can form up to 8 or 9 hydrogen bonds. Therefore, the likely reason for the increased binding ability of IDA are the hydrophobic interactions with the inner APO surface. This type of interaction of IDA may be enhanced because of the protrusions of aromatic rings of amino acids to the inner space of APO cage, for such amino acids as phenylalanine (Phe), tryptophan (Trp), and tyrosine (Tyr). The hydrophobicity of these amino acids increases in order: Tyr < Trp < Phe, according to the Wimley–White interfacial hydrophobicity scale for amino acids [60] that combines the effects of the side chain and amide group. Also, some aliphatic amino acids show hydrophobic properties that increase in order: Ala < Pro < Met < Val < Leu < Ile [60]. They may also interact with IDA. The hydrophobic interactions between APO and IDA are consistent with our results obtained with circular dichroism spectroscopy (Figure 3) showing that upon the addition of IDA, the α-helix content in APO decreased by 2.64% indicating the involvement of IDA in binding to the APO’s amino acid residues forming the α-helix.

(iii) The interactions (i) and (ii) are superimposed on lipophilicity-independent specific interactions of anthracyclines with binding sites on the inner surface of APO, referred to by Kilic et al. [30] as the complex formation APO(DOX*_n_*) with *n* = 24 ± 3. Unlike in the case of cisplatin binding to APO, with the formation of APO(cisplatin_n_) complex, where the metal atom actually interacts with nitrogen free electron pair of His 133 of APO, the interactions of anthracyclines with APO are due to the supramolecular interactions involving multiple hydrogen bonds at the biorecognition site for ANTR triple ring on the inner APO surface. The ANTR drugs can form up to nine hydrogen bonds with APO. The molecular ratio of APO:drug = 1:24 can be compared with the theoretical values for non-specific close-packing adsorption of a model ANTR derivative, DOX, on the inner APO surface, ranging from 150 to 308 DOX molecules per APO nanocage, depending on the drug molecule orientation (Table 3). On the other hand, the number of free ANTR molecules entrapped in the APO cage volume from a solution during the encapsulation process is less than one, when the ANTR concentration of 1 mM, used in this work, is taken into account. Based on this analysis, the contribution of supramolecular binding of ANTR drugs to the total encapsulation number is between 66 and 100%. The nonspecific adsorption, due to the polar and hydrophobic interactions, contributes up to ~34% and the ANTR trapping in nanocage volume less than 1%, unless the aggregation of the entrapped drug molecules is induced, e.g., by metal ion bridging, binding to DNA or negatively charged polymers like polylactic acid.

(iv) A considerable number of drug molecules can also be bound to the external surface of an APO. For instance, in the case of a non-specific adsorption of DOX in side-on orientation, the maximum surface excess of DOX is found to be 693 molec/APO on the outer APO surface, compared to 308 molec/APO on the inner APO surface (Table 3). However, due to the thorough washing and centrifugation of the nanodrugs (five times each preparation), the drugs bound reversibly to the outer APO surface were successfully removed and the number of residual drug molecules was negligible or small. From the release kinetics data of Figure S1 it can be seen that the drugs released in first minutes of the release, which are due to the release from the outer APO surface, were 0–0.2% and 0.5 ± 0.2%, for DOX and EPI, respectively, and 2.5 ± 0.4% for IDA. Therefore, in this work, we have excluded the contribution of the drugs bound on the outer APO surface from further consideration.

Targeting of tumor cells has been achieved through the known ability of APO to recognize the receptors: TfR1 and SCARA5, which are overexpressed in rapidly proliferating cells, such as cancer cells [19,36], and through the recognition of FR receptors by the folate moiety attached to the APO nanocarriers. The structures of APO-TfR1 and APO-SCARA5 are not well understood as yet. According to Li et al. [19], upon receptor-mediated endocytosis, APO enters endosomes and lysosomes where it is disassembled due to the low pH. However, according to Zhang et al. [15], APO may be translocated directly to the nucleus where it is degraded releasing the drug payload. On the other hand, FR overexpressed in cancer cells, binds folates with a very high affinity (*K*_d_ = 1 ÷ 10 nM), to absorb folate-coated APO nanocarriers via receptor-mediated endocytosis [13,14].

In vitro evaluation of the APO(drug) activity in MCF10A, MDA-MB-231, and HeLa cells, using flow cytometry and cytotoxicity/growth inhibition assays, performed in this work, show a reduced drug delivery rate with APO(drug) nanodrugs in comparison to the free drugs. This is expected due to the slow APO nanocage opening during the drug release phase. These measurements also corroborate the high apoptotic activity of the APO(ANTR) nanodrugs against cancer cells: MDA-MB-231 adenocarcinoma and HeLa cervical cancer cells, with APO(IDA) showing the highest efficacy by generating 82% apoptotic cells in MDA-MB-231 cell line at 1 µM IDA encapsulated in APO(IDA). This contrasts with lower apoptotic activity of the nanodrugs against healthy MCF10A epithelial cells, demonstrating selective cytotoxicity against targeted cancer cells. This means that folate-enhanced APO(ANTR) nanocarriers offer targeted drug delivery and can mitigate drug side effects, reduce damage to healthy cells, and hold a potential to overcome multiple drug resistance.

### 3.2. Model Calculations for ANTR Drugs Encapsulation in APO Nanocages

Based on the discussion presented in the previous section, we have developed a model for anthracycline drugs encapsulation in APO nanocages, based on the Gibbs free energies for the following interactions:

(1) ∆G^0^_pol_ due to polar interactions leading to the formation of hydrogen bonds and immobilization of ANTR molecules on the inner surface of an APO cage on hydrophilic sites;
(2) ∆G^0^_hph_ due to hydrophobic interactions leading to the immobilization of ANTR on the inner APO cage surface on hydrophobic sites;
(3) ∆G^0^_ens_ due to the supramolecular ensemble formation APO(ANTR*_p_*), with the maximum encapsulation number *P* = *n*_ens,max_ = 24;
(4) ∆G^0^_trap_ due to the entrapment of free ANTR drug molecules in the inner volume of an APO nanocage;
(5) ∆G^0^_el_ due to electrostatic attraction of positively charged ANTR molecules, at pH < pK_a_ of the protonation of the -NH_2_ moiety, and negatively charged dissociated local carboxyl groups of APO. The point of zero charge (pzc) for APO is: pzc = 4.4 [61,62]). The electrostatic interactions are mainly acting during the encapsulation and drug release processes, in acidic environments.

The total Gibbs free energy for ANTR encapsulation is given by:

(1) $$\Delta G_{\mathrm{tot}}^{0}=\sum_{i}\Delta G_{i}^{0}=\Delta G_{\mathrm{pol}}^{0}+\Delta G_{\mathrm{hph}}^{0}+\Delta G_{\mathrm{ens}}^{0}+\Delta G_{\mathrm{trap}}^{0}+\Delta G_{\mathrm{el}}^{0}$$

where ∆G^0^_trap_ = 0, unless a mechanism for linking ANTR molecules together is provided, e.g., by bridging via metal ions. The encapsulation number *n* is defined here as the total number of drug molecules bound to the inner APO surface and trapped in the inner space of the APO cage.

Among the processes listed above, process (3) has recently been recognized and well described in the literature [30]. Due to the supramolecular binding, it confers the highest binding constant and thus, the binding sites on inner APO surface for the ensemble APO(ANTR_24_) are filled before other sites available on APO surface are filled in processes (1) and (2). The contribution of the entrapment (4) to the encapsulation under conditions of our experiments, is less than 1 drug molecule per APO nanocage. It has to be emphasized that a considerable increase of the drug concentration in the encapsulation solution, to increase the drug entrapment rate, is limited due to the relatively low solubility of the ANTR derivatives. The increased entrapment can in principle be achieved by providing a mechanism for linking ANTR molecules together in the APO inner solution, for instance by Fe(III)-bridging [63]. An attempt made to bind protonated daunomycin to negatively charged poly-L-aspartic acid resulted in the increased entrapment by ca. 0.22 molecules of DAU/APO [42]. Unfortunately, not much attention has been paid to processes (1) and (2), which require further elucidation. These processes appear to be competitive with each other and we have found that their contributions to the drug encapsulation in APO is considerable. Indeed, the increase of hydrophobic interactions is found for drugs with high lipophilicity, as expected. On the other hand, the increased dipolar interactions with the formation of hydrogen bonds are seen for drugs with low lipophilicity. The main contributions to the drug encapsulation number *n*_tot_ are:

(2) $$\begin{array}{l} n_{\mathrm{tot}}^{}=\sum_{i}n_{i}^{}=n_{1}^{}+n_{2}^{}+p+n_{\mathrm{trap}}^{}+n_{\mathrm{el}}^{}\\ n_{1,\max}=f(P),n_{2,\max}=f(P);\\ n_{\mathrm{ens},\max}=24,\;\mathrm{for}\;C_{D}=\infty ;\\ n_{1,\max}=n_{1,\max}^{0}\;,\;n_{2,\max}=n_{2,\max}^{0}\;,\;\mathrm{for}\;P=0;\\ n_{1,\max}=n_{1,\max}^{\infty }\;,\;n_{2,\max}=n_{2,\max}^{\infty }\;,\;\mathrm{for}\;P>>15; \end{array}$$

The model calculations for competing processes (1) and (2) are described below.

(1) *Binding of ANTR drugs on non-hydrophobic sites of APO*.

The immobilization of a drug molecule D on a free non-hydrophobic binding site on the inner APO surface □_APO,1_ can be expressed by the reaction:

D   +   □_APO,1_   =   D_APO,1_ *C*_D_  (*n*_1,max_−*n*_1_)C_APO_  *n*_1_*C*_APO_ (3)

where *C*_D_ is the drug concentration in solution, *C*_APO_ is the concentration of APO nanocarriers in solution, *n*_1_ is the number of drug molecules already bound on hydrophobic sites of the inner APO surface, and *n*_1,max_ is the maximum number of available non-hydrophobic sites for drug binding per APO molecule. The binding equilibrium constant *K*_1_ is given by:

(4) $$K_{1}=\frac{n_{1}C_{\mathrm{APO}}}{\left(n_{1,max}-n_{1}\right)C_{\mathrm{APO}}C_{D}}$$

and the Gibbs free energy by:

(5) $$\Delta G_{1}^{0}=-RT\ln\left(K_{1}\right)$$

In these equations, *n*_1,max_ and *K*_1_ depend on *P*. Since *n*_1,max_ evidently decreases with increasing lipophilicity *P*, we assume a simple relationship:

(6) $$n_{1,\max}=n_{1,\max}^{0}+a_{1}P$$

with the slope: *a*_1_ < 0. By combining Equations (4) and (6), after some algebra, one obtains:

(7) $$n_{1}=\frac{K_{1}C_{D}\left(n_{1,\max}^{0}+a_{1}P\right)}{\left(1+K_{1}C_{D}\right)}$$

Due to the low internal surface coverage of APO by the drug molecules, the lateral interactions between immobilized drug molecules are expected to be rather weak, especially since the number of drug molecules bound to hydrophilic sites on APO decreases with increasing *P*. We assume that the effect of *P* on *K*_1_ follows a linear dependence since *P* is a simple distribution ratio of the drug between a hydrophobic (octanol) and a hydrophilic (aqueous PBS buffer) phase:

(8) $$K_{1}=K_{1}^{0}+b_{1}P$$

where *b*_1_ < 0. Therefore, the equation for *n*_1_ becomes:

(9) $$n_{1}=\frac{C_{D}\left(K_{1}^{0}+b_{1}P\right)\left(n_{1,\max}^{0}+a_{1}P\right)}{1+C_{D}\left(K_{1}^{0}+b_{1}P\right)}$$

However, this function has a singularity at *P* given by the equation:

(10) $$P=\frac{-C_{D}K_{1}^{0}-1}{b_{1}C_{D}}$$

where it becomes indeterminate. To circumvent the problem of singularity, two other approaches have been explored, both based on the loss function, $n_{1}^{\mathrm{loss}}$, representing the number of lost binding sites with increasing *P*, using:

(i) a hyperbolic averaging for the number of sites lost with increasing *P* and the number of residual sites *n*_1,res_, which remain available even at the highest *P* due to the characteristic properties of the ANTR triple ring structure, or
(ii) a loss function representing the number of blocked hydrophilic sites, which increases with increasing lipophilicity *P*, according to the phenomenological equation:

(11) $$n_{1}^{\mathrm{loss}}=\frac{n_{1,\max}^{\mathrm{loss}}K_{bl}}{1+K_{bl}}$$

where *K*_bl_ is the ratio of the number of blocked sites to the number of free hydrophilic sites and $n_{1,\max}^{\mathrm{loss}}$ is the maximum number of sites that can be lost while the triple ring structure characteristic of ANTR derivatives is still maintained. The residual number of hydrophilic sites, which remains intact even at high lipophilicity of ANTR derivatives, is given by:

(12) $$n_{1,\mathrm{res}}^{}=n_{1,\mathrm{tot}}^{}-n_{1,\max}^{\mathrm{loss}}$$

where $n_{1,\mathrm{tot}}^{}=n_{1}^{0}$, i.e., the number of hydrophilic sites at *P* = 0.

The constant *K*_bl_ represents the process of hydrophilic sites blocking and its value is associated with increasing lipophilicity *P* of the ANTR derivatives. We assume a simple relation: *K*_bl_ *= m*_1_*P*, where *m*_1_ > 0 since blocking increases with lipophilicity. Thus:

(13) $$n_{1}^{\mathrm{loss}}=\frac{n_{1,\max}^{\mathrm{loss}}m_{1}P}{1+m_{1}P}$$

It is seen from this equation that $n_{1}^{\mathrm{loss}}$ increases from 0 to $n_{1,\max}^{\mathrm{loss}}$ when *P* increases from 0 to infinity. After a simple transformation, including reflection of the loss function with respect to the abscissae and shifting upwards by $n_{1}^{0}$, one obtains the expression for actual contribution of hydrophilic sites *n*_1_ to the encapsulation excess as a function of *P*:

(14) $$n_{1}=n_{1}^{0}-n_{1}^{\mathrm{loss}}$$

(15) $$n_{1}=n_{1}^{0}-\frac{n_{1,\max}^{\mathrm{loss}}m_{1}P}{1+m_{1}P}$$

**Figure 8:**
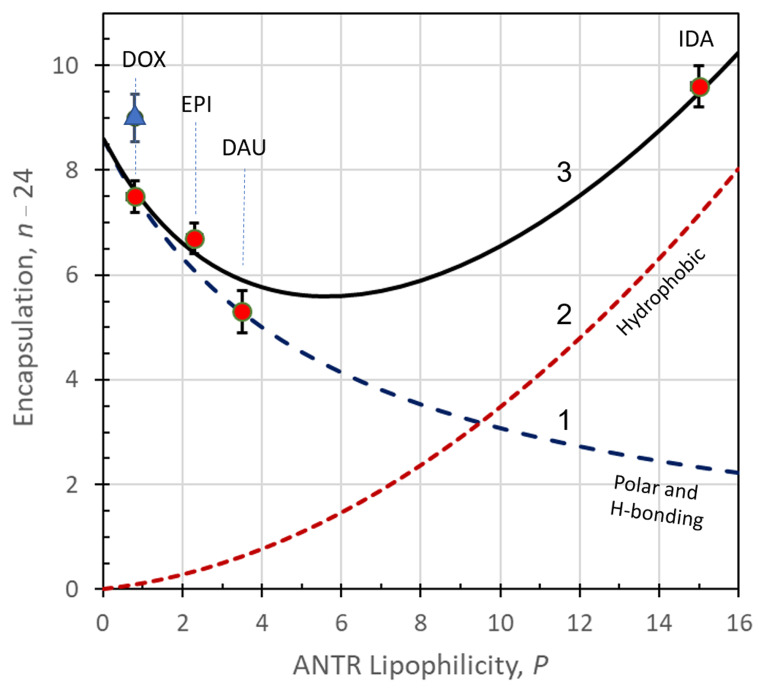
Model calculations for ANTR drugs encapsulation in APO nanocage carriers: (1) contribution of dipolar and hydrogen binding, (2) contribution of hydrophobic interactions, (3) sum of contributions (1) and (2); experimental points are shown with markers: circles (this work), triangle (according to [18]).

This dependence of the encapsulation excess *n*_1_ on *P*, showing a decrease in dipolar interactions and H-bonding with increasing lipophilicity is plotted in Figure 8, curve (1).

For the limiting conditions, we have:

(16) $$(a)\;n_{1}=n_{1}^{0},\;\mathrm{for}\;P=0$$

(17) $$(b)\;n_{1}=n_{1}^{0}-n_{1.\max}^{\mathrm{loss}}=n_{1,\mathrm{res}},\;\mathrm{for}\;P>>15$$

While both approaches, (i) and (ii), lead to satisfactory results for intermediate and large values of *P*, the approach (i) fails at *P* values close to 0, showing errors that depend on the parameters involved. Therefore, approach (ii), based on the loss function for the hydrophilic sites, has been used in further analysis.

(2) *Binding of ANTR drugs on hydrophobic sites of APO*

While the anthracycline drugs with high lipophilicity still contain the basic triple ring with many of the original polar functional groups, found in DOX, and are still able to form H-bonding and undergo polar interactions, the dominating contribution to the ANTR binding to APO nanocages is now due to the hydrophobic interactions APO-ANTR. These interactions increase with increasing lipophilicity, as expected, whereas the dipolar interactions and hydrogen bonding are being diminished toward the minimum required by the structural definition of anthracyclines family.

For hydrophobic binding, expressed by the reaction:

D   +   □_APO,2_   =   D_APO,2_ *C*_D_  (*n*_2,max_ − *n*_2_)C_APO_  *n*_2_*C*_APO_ (18)

the equilibrium constant *K*_2_ is given by:

(19) $$K_{2}=\frac{n_{2}C_{APO}}{\left(n_{2,max}-n_{2}\right)C_{APO}C_{D}}$$

and the Gibbs free energy by:

(20) $$\Delta G_{2}^{0}=-RT\ln\left(K_{2}\right)$$

We assume that the maximum number of available sites for binding, *n*_2,max_, increases linearly with increasing lipophilicity:

(21) $$n_{2,\max}=n_{2,\max}^{0}+a_{2}P$$

Therefore, in contrast to Equation (5), *a*_2_ is now positive since more hydrophobic sites appear in drug molecules with increasing *P*. By combining Equations (19) and (21), one obtains:

(22) $$n_{2}=\frac{K_{2}C_{D}\left(n_{2,\max}^{0}+a_{2}P\right)}{\left(1+K_{2}C_{D}\right)}$$

The value of *K*_2_ also depends on *P*. We assume a simple linear dependence:

(23) $$K_{2}=K_{2}^{0}+b_{2}P$$

with *b*_2_ > 0. Therefore, the equation for *n*_2_ becomes:

(24) $$n_{2}=\frac{C_{D}\left(K_{2}^{0}+b_{2}P\right)\left(n_{2,\max}^{0}+a_{2}P\right)}{1+C_{D}\left(K_{2}^{0}+b_{2}P\right)}$$

resembling the form of Equation (8), but here the terms *a*_2_*P* and *b*_2_*P* are positive and there is no singularity in the range of *P* accessible experimentally, i.e., from *P* = 0 to 15. The contribution *n*_2_ to *n*, given by this equation, is plotted in Figure 8, curve (2).

For limiting conditions, we have:

(25) $$(a)\;n_{2}=n_{2,\max}^{0}\frac{K_{2}C_{D}}{\left(1+K_{2}C_{D}\right)},\;\mathrm{for}\;P=0$$

(26) $$(b)\;n_{2}=a_{2}P,{\;\mathrm{for}\;a}_{2}P>>n_{2,\max}^{0}$$

The sum of contributions *n*_1_ and *n*_2_ is represented in Figure 8 by curve (3) which fits well to the experimental data shown as circles. Representative data from literature references are shown as triangles (data of Liang et al. [18]).

The fitting performed provides the following values of constants:

(27) $$n_{1}^{0}=8.6;\;n_{1,\mathrm{res}}^{}=0.5;\;a_{1}=-2;$$

(28) $$n_{2,\max}^{0}=0;\;a_{2}=18.9;\;K_{2}^{0}=440{\;M}^{-1};\;b_{2}=138;\;C_{D}=1.03\times 10^{-5}\;M$$

With the parameters shown in Equations (24) and (25), the number of hydrophobic sites spans from 0 to 283.5 per APO, for *P* changing from 0 to 15, close to the maximum monolayer coverage of ANTR drugs per inner APO surface in vertical orientation: 308.3 − 24 = 284.3 (see: Table 3).

### 3.3. Performance of APO(ANTR) Nanodrugs in Treatment of HeLa and MDA-MB-231 Cancer Cell Lines and Non-Tumorigenic MCF10A Cells In Vitro

The experiments performed indicate clearly that the activity of ANTR derivatives, encapsulated in APO nanocage carriers (i.e., the nanodrugs), is lower than that of free ANTR drugs. This behavior is expected due to relatively slow drug release from the nanocarriers in comparison to the immediate drug availability in the case of free drugs. This is corroborated by monitoring of the drug release from APO nanocages. In fact, the drug release transients, recorded for APO(ANTR) nanocarriers for all ANTR drugs under study in buffer solutions with pH: 7.4, 5.6, 4.6, and 3.6 (SI, Figure S1), clearly show that the rate of drug release from APO nanocages is relatively slow and further decreases with increasing pH of the medium. Another proof of the lower activity of drugs encapsulated in APO nanocages with respect to free ANTR drugs, comes from the flow cytometry apoptosis profiles (Figure 4 and Figure 5), which shows that the fractions of late apoptotic cells for MDA-MB-231 breast cancer cells observed for all four nanodrugs are smaller than those for free drugs. Moreover, the cell survival/cytotoxicity assays performed with three cell lines treated with APO(IDA) and free IDA (Figure 6) also corroborate lower activity of ANTR nanodrugs in comparison to free ANTR drugs. Therefore, the anticipated lower activity of ANTR drugs, encapsulated in APO nanocage carriers, has been confirmed for in vitro experiments in buffer solutions.

The advantage of using APO-based drug nanocarriers for targeted CDD is their ability to recognize cancer cells and deliver the drug payload selectively to cancer cells. The APO nanocarriers, utilized in this work can directly interact with the TfR1 and SCARA 5 receptors [19,36] overexpressed in cancer cells. We have found that the treatment of MDA-MB-231 adenocarcinoma cells and HeLa cervical cancer cells with free IDA and APO(IDA) nanocarriers (Figure 6D) resulted in the death of 86 ± 3% and 93 ± 2% of cancer cells, respectively, while only 59 ± 8% dead cells were observed for non-tumorigenic MCF10A cells, corroborating the high selectivity of nanodrugs in targeting cancer cells. In addition to that, functionalization of the external APO surface with folate ligands enables binding of nanocarriers to FR receptors in the membrane of cancer cells [16,57]. The tests performed with and without folate ligands immobilized on APO indicated that the presence of the folates (confirmed with FTIR spectra, Figure 3D) does not interfere with the drug release tested at pH 7.4 and 4.6 (Figure S2).

The drug release transients, presented in Figure S1, for ANTR derivatives in solutions with pH varying in the range from: 7.4 to 3.6, indicates the increasing release rates with decreasing pH of the medium. It is very likely that the complex intracellular conditions, including local pH, proteolytic environment, and other factors, may further influence the drug release rate. Future studies involving drug release kinetics under simulated intracellular conditions will bring new insights into the mechanisms and kinetics of drug release from APO(drug) nanocarriers in CDD.

In summary, an extensive cytotoxicity testing has been performed to evaluate the in vitro activity of novel anthraquinone nanodrugs toward cancer cell lines (HeLa cervical cancer cells and MDA-MB-231 adenocarcinoma breast cancer cells), as well as the non-tumorigenic cell line (MCF10A epithelial cells). The characterization of drug activity included determination of the dose–response curves for free ANTR drugs and APO-encapsulated nanodrugs for MDA-MB-231 cells, flow cytometry tests to determine the extent of apoptosis induced by the nanodrugs under study, cytotoxicity/growth inhibition tests, as well as the determination of EC_50_ values for the nanodrugs. The cytotoxicity tests have demonstrated the high activity of APO-encapsulated ANTR nanodrugs toward the cancer cell lines and support the proposed model of ANTR drugs encapsulation. This model is based on evaluation of the effects of different kinds of interactions that influence the encapsulation excess of ANTR drugs in APO nanocage nanocarriers. The proposed encapsulation model may serve not only to assess the in vitro activity of the APO-based nanodrugs but can also help in studying other novel theranostic drug nanocarriers. The use of nanocarriers is now becoming a necessity due to the impasse in further development of systemic chemotherapies due to adverse side effects and collateral damage to healthy cells, often leading to the organ failure and even death. We hope that the model proposed here can be in future adopted also to other drug nanocarriers and can serve in the development of controlled drug delivery systems for successful cancer treatment.

## 4. Materials and Methods

### 4.1. Chemicals

The anticancer drugs, epirubicin (EPI), idarubicin (IDA), and daunorubicin (DAU), were received from Selleckchem (Houston, TX, USA), and doxorubicin (DOX) was purchased from Sigma-Aldrich Co. (St Louis, MO, USA); the drugs were used as received. The apoferritin from equine spleen (APO) was purchased from Sigma-Aldrich Co. (St Louis, MO, USA). N-(3-dimethylaminopropyl)-N′-ethylcarbodiimide hydrochloride (EDC), N-hydroxysuccinimide (NHS), folic acid, and other chemicals were obtained from Aldrich Chemical Co. (Milwaukee, WI, USA) and were of analytical grade and used as received. Phosphate-buffered saline (PBS) pH 7.4 consisted of 1.8 mM Na_2_HPO_4_, 137 mM NaCl, 2.7 mM KCl, and 10 mM KH_2_PO_4_. McIlvain buffer (MI buffer) solutions (pH 3.6, 4.6, and 5.6) were prepared from 0.2 M Na_2_HPO_4_ and 0.1 M citric acid stock solutions. All aqueous solutions were prepared with deionized water (resistivity of 18.2 MΩ cm) purified with a Milli-Q reagent grade water system (Merck Millipore, Billerica, MA, USA).

### 4.2. Apparatus

The fluorescence spectra were recorded using LS55 Spectrometer (Perkin Elmer, Waltham, MA, USA.) equipped with 20 kW Xenon light source operating in 8 μs pulsing mode. Separate monochromators for the incident and detector beams enabled use of monochromatic radiation with wavelengths from 200 to 700 nm. The dual detector system consisted of a photomultiplier tube (PMT) and an avalanche photodiode. The UV–Vis spectra were recorded using a Varian Cary 50 Bio UV–Visible Spectrophotometer (Agilent Technologies, Santa Clara, CA, USA) in the range from 200 nm to 700 nm at room temperature. FTIR spectra of APO nanocages were obtained using Model Nicolet iS-10 FTIR instrument (Thermo Fisher Sci., Waltham, MA, USA) working in specular mode enabling the reflection from the sensor surface to be analyzed. The circular dichroism (CD) spectra were recorded on a J-715 spectropolarimeter (JASCO, Tokyo, Japan). Far-UV (190–240 nm) recordings were performed in a 0.5 cm pathlength quartz cuvette.

### 4.3. Software

The model calculations of contributions of different kinds of interactions between APO nanocage carriers and ANTR drugs were performed using digital simulation and fitting utilities in Microsoft EXCEL software and ORIGIN v. 9.1 data analysis software (Origin Lab Corp., Sunnyvale, CA, USA). All statistical functions were calculated using ORIGIN. The dose–response relations and EC_50_ values for free drugs and APO-encapsulated nanodrugs were also obtained using ORIGIN software package.

### 4.4. Incorporation of Anthracycline Drugs to Apoferritin Nanocages

The four anthracyclines studied have a similar structure and their main pK_a_ values are close to 8.46 and 7.34, due to the amine group protonation and phenolic dissociation, respectively. Hence, the net charges carried by these anthracycline molecules do not differ significantly. However, the small structural differences, including additional -O-CH_3_ group at C4, as well as -CH_2_OH or -CH_3_ group at C13, result in considerable differences in drugs lipophilicity and supramolecular interactions with apoferritin protein, thus influencing the encapsulation efficiency. The measurements performed in this work were designed to evaluate the efficiency of encapsulation of anthracyclines under study in APO nanocages.

The encapsulation of anthracycline drugs in APO nanocages was carried out by the disassembly/reassembly protocols based on those developed recently [39,40,43,44] and with changes adopted for the drugs used in this study. Briefly, 5 or 25 µL of 100 mg/mL horse spleen APO (final concentration of APO 1 or 5 mg/mL) was mixed with 10 or 50 µL of 10 mM anthracycline derivative (DOX, EPI, DAU and IDA) in a molar ratio of APO:drug of 1:100. The total volume of the mixtures was 500 µL which was obtained by adding appropriate amounts of Milli-Q water. A 4 μL aliquot of 1 M hydrochloric acid was added to decrease the solution pH to 2.0 and dissociate the APO. Then the pH value was maintained for 15 min. Afterward, the pH was slowly increased up to 7.4 using 4 µL of 1 M sodium hydroxide. The resulting solution was stirred under room temperature for 2 h to encapsulate the anthracycline in the APO. Then, the mixture was rinsed five times with Milli-Q water using Amicon Ultra-0.5 mL 30K (Merck Millipore, Billerica, MA, USA) to remove unbound drug molecules. The obtained anthracycline-containing APO nanocages (APO(drug)) were stored at 4 °C. The stability of APO(ANTR) nanodrugs was tested by storing them at 4 °C for 30 days. The drug activity was found to decrease by 5.7% (*n* = 5). For all experiments performed in this work, freshly prepared APO(ANTR) nanodrugs were used.

### 4.5. Modification of APO(Drug) Nanocages with Folic Acid

To improve the effectiveness of APO(drug) nanocages in targeted drug delivery, we applied covalent binding of folic acid (FA) to an APO(drug) nanocage using a modification of the standard EDC/NHS reaction. We dissolved 10 mM FA in 5% NaHCO_3_. To activate carboxyl groups of the folic acid, 10 µL of FA solution was added to 900 µL of EDC/NHS mixture containing 7 mM EDC and 7 mM NHS in water and the resulting solution was allowed to react at room temperature for 30 min. The activated mixture was added to the APO(drug) nanocages, prepared earlier by centrifugation using Amicon Ultra-0.5 mL 3K (Thermo Fisher Sci., Waltham, MA, USA) to remove water, and incubated overnight at room temperature. The APO(drug)@FA nanocarriers were purified through the Amicon Ultra-0.5 mL 30K and washed 3 times with Milli-Q water. The obtained product was dispersed in water for further characterization and application.

### 4.6. Encapsulation Ratio Drug:APO

The quantification of encapsulated anthracyclines was performed by determining the concentration of free drugs in solution, separated from APO(drug) NPs by filtration and the initial drug concentration in the encapsulation solution. The concentrations of free drugs were evaluated using fluorescence emission measurements, for *λ*_ex_ = 480 nm. All the data are expressed as the average of at least three determinations.

### 4.7. Releasing of Anthracyclines form APO(Drug) Nanocages

The kinetics of drug release from APO(drug) was evaluated using PBS buffer pH 7.4 and McIlvain buffers with pH 3.6, 4.6, and 5.6. In brief, 300 µL of the appropriate buffer was added to the APO(drug) and samples were incubated for 6 h at 37 °C. At the pre-set time points (1 h apart), the solution with unbound drug was separated by filtration using Amicon-Ultra-0.5 mL 3K at RT. The container with APO(drug) nanocarriers was replenished with an equal volume of blank buffer (300 µL). The release study was carried out in triplicate for each pH.

### 4.8. Cell Culture

Cells were cultured in a humidified incubator at 37 °C with 5% CO_2_. The cell lines used in this study: standard HeLa cancer cells, MDA-MB-231 human mammary gland breast cancer adenocarcinoma cells, and MCF10A human mammary non-tumorigenic epithelial cells, were obtained from American Type Culture Collection (Manassas, VA, USA). HeLa and MDA-MB-231 cells were cultured as monolayers in Dulbecco’s Modified Eagle Medium (DMEM) supplemented with 10% fetal bovine serum (FBS). The growth medium for MCF10A cells contained DMEM, horse serum (5%), epidermal growth factor (EGF, 20 ng/mL), hydrocortisone (0.5 mg/mL), cholera toxin (100 ng/mL), and insulin (10 μg/mL). Cells were routinely passed at 80–90% confluence, using trypsin/EDTA.

### 4.9. Cell Death Assay

To determine the fraction of dead and apoptotic cells induced by targeted free anthracyclines (DOX, EPI, IDA) and APO nanocages uploaded with encapsulated anthracyclines (DOX, EPI, or IDA), the Annexin V method was used. MDA-MB-231 cells were exposed to the indicated concentrations of anticancer drugs and APO nanocages for 72 h. Media and cells were then collected, pelleted, and processed according to the Muse™ Cell Analyzer Annexin V and Dead Cell Kit instructions (MCH100105). Briefly, 100 µL of cells in suspension were added to tubes. Next, 100 μL of the Muse™ Annexin V & Dead Cell Reagent was added to each tube. Samples were stained for 20 min at room temperature in the dark. Approximately 5000 cells were gated for analysis per sample; triplicate biological replicates were analyzed.

### 4.10. Cytotoxicity/Growth Inhibition Tests by Crystal Violet Staining

Cytotoxicity/growth inhibition assay is an in vitro cell survival assay based on the ability of a single cell to grow into a colony. HeLa, MDA-MB-231, and MCF10A cells were seeded onto six-well plates at a concentration of 150,000 cells/well such that they were 40% confluent at 24 h. The cells were then treated with IDA and APO(IDA) at concentrations: 0.0, 0.5, and 1.0 µM of the drug. After the treatment, cells were incubated in 5% CO_2_ atmosphere at 37 °C for 72 h to allow for colony formation and growth. After the 72-h treatment, pictures of each well on the six-well plates were taken. The cells were then fixed in 3.7% formaldehyde for 15 min. The fixed cells were rinsed with PBS and stained for 10 min using 0.1% crystal violet. After staining, the plates were rinsed with water to remove the staining solution and allowed to air dry. To quantify, the cells were solubilized in 1% SDS and the absorbance at 590 nm was measured. The percentage of cell survival (PCS) was calculated as follows:

(29) $$PCS=\frac{\;\mathrm{No}.\;\mathrm{of}\;\mathrm{cells}\;\mathrm{after}\;\mathrm{treatment}}{\mathrm{No}.\;\mathrm{of}\;\mathrm{cells}\;\mathrm{in}\;\mathrm{UC}}\times 100$$

where UC stands for the untreated control.

## Figures and Tables

**Table 1:** The encapsulation excess *n* for ANTR derivatives in APO nanocages, obtained using encapsulation solutions of 1 mg/mL of APO with ANTR:APO molar ratios of 100:1.

Anthracycline	Encapsulation Excess *, *n* (ANTR:APO)	Drug Lipophilicity ** P_drug_
Doxorubicin	31.5 ± 0.6	0.8 ± 0.1
Epirubicin	30.7 ± 1.0	2.3 ± 0.3
Daunorubicin	29.3 ± 1.0	3.5 ± 0.5
Idarubicin	33.6 ± 0.7	15.0 ± 1.3

* This work; ** Drug lipophilicity defined as the octanol/PBS partition coefficient for anthracycline drugs (Ref. [8]).

**Table 2:** Half-maximal effective concentrations EC_5_ = for free ANTR drugs and APO-encapsulated ANTR nanodrugs, obtained for in vitro treatment of MDA-MB-231 breast cancer cells.

ANTR Drug	EC_50_ for Free Drug nM	EC_50_ for APO(Drug) nM
Doxorubicin	68.0	224.9
Epirubicin	39.8	289.8
Idarubicin	14.1	92.5

**Table 3:** Comparison of experimental DOX encapsulation excess *n* for APO(DOX_n_) nanocarriers * with theoretical data for nonspecific adsorption of DOX on inner APO surface at maximum coverage.

Property	Inner APO Surface	Comments	Reference
APO surface area	201.0 nm^2^	*r*_int_ = 4 nm, *r*_ext_ = 6 nm	this work
*n*_DOX_/APO, horizontal	196	theor., non-spec. ads. **	this work
*n*_DOX_/APO, vertical	308	theor., non-spec. ads.	this work
*n*_DOX_/APO, side-on	150	theor., non-spec. ads.	this work
*n*_DOX_/APO, exprtl.	24 ± 3	APO-DOX complex	[30]
*n*_DOX_/APO, exprtl.	33 ***	upload in 8 M urea	[18]
*n*_DOX_/APO, exprtl.	31.5 ± 0.6	−	this work
*n*_IDA_/APO, exprtl.	33.6 ± 0.7	IDA drug	this work
*n*_cisPt_/APO, exprtl.	21.6–55.2	cisplatin drug	[38]
*n*_cisPt_/APO, exprtl.	15	cisplatin drug	[29]

*—APO disassembly and drug upload at pH ~2 unless otherwise stated; **—non-spec. ads. relates to the nonspecific adsorption of drug on the inner APO surface assuming maximum packing density for the drug orientation specified in first column; calculations based on DOX dimensions: *a* = 1.45 nm, *b* = 0.705 nm, *c* = 0.925 nm, estimated from the electronic structure of DOX; nonspecific adsorption of drug on the external APO surface is negligible due to nanocarrier washing and removal of free drugs from the solution; ***—after APO disassembled in 8 M urea.

## Supplementary Materials

The following are available online at https://www.mdpi.com/1422-0067/22/3/1362/s1, Figure S1: Temporal evolution of anthraquinone drugs’ release from APO nanocages of different pH, Figure S2: Temporal evolution of DOX release at pH 4.6 and 7.4 from APO(DOX): bare and modified with folic acid.

## Abbreviations

ANTR	Anthracycline
APO	Apoferritin
CD	Circular dichroism
CDD	Controlled drug delivery
DAU	Daunorubicin
DOX	Doxorubicin
DMEM	Dulbecco’s Modified Eagle Medium
EDC	N-(3-dimethylaminopropyl)-N′-ethylcarbodiimide hydrochloride
EGF	Epidermal growth factor
EPI	Epirubicin
FA	Folic acid
FBS	Fetal bovine serum
FR	Folate receptor
GSH	Glutathione
IDA	Idarubicin
MDR	Multiple drug resistance
MI buffer	McIlvain buffer
NHS	N-hydroxysuccinimide
NP	Nanoparticle
PBS	Phosphate-buffered saline
PCS	Percentage of cell survival
PMT	Photomultiplier tube
pzc	Point of zero charge
ROS	Reactive oxygen species
SCARA5	Scavenger Receptor Class A Member 5
TDD	Targeted drug delivery
TfR1	Transferrin receptor 1
