# Supplementary material for: Cancer-Targeted Controlled Delivery of Chemotherapeutic Anthracycline Derivatives Using Apoferritin Nanocage Carriers
Source: Int J Mol Sci. 2021 Jan 29;22(3):1362. doi: 10.3390/ijms22031362 (PMC7866407; doi:10.3390/ijms22031362)
Supplement: Supplementary file 1 [file ijms-22-01362-s001.pdf]

ORIGINAL RESEARCH

Supplementary Materials

Cancer-targeted controlled delivery of chemotherapeutic anthracycline derivatives using apoferritin nanocage carriers

Katarzyna Kurzątkowska[1,2]*, Manuel A. Pazos II [3], Jason I. Herschkowitz[3], Maria Hepel[1]*

[1] Department of Chemistry, State University of New York at Potsdam, Potsdam, NY 13676, USA
[2] Department of Biosensors, Institute of Animal Reproduction and Food Research, Polish Academy of Science, Tuwima 10 Str., 10-748 Olsztyn, Poland
[3] Department of Biomedical Sciences, Cancer Research Center, University at Albany, State University of New York, Rensselaer, NY 12222, USA

* *Corresponding authors:*
K. Kurzątkowska (k.kurzatkowska@pan.olsztyn.pl),
M. Hepel (hepelmr@potsdam.edu)

Correspondence:

Maria Hepel,

Department of Chemistry, State University of New York at Potsdam, Stowell Hall,

44 Pierrepont Ave., Potsdam, NY 13676, USA

Tel: +1.315.267.2267,

Fax: +1.315.267.3170,

Email: hepelmr@potsdam.edu

Methods

Chemicals

The anticancer drugs (epirubicin – EPI, idarubicin – IDA, and daunorubicin – DAU) were received from Selleckchem (Houston, TX, USA), and the anticancer drug, doxorubicin (DOX), was purchased from Sigma-Aldrich Co. (St Louis, MO, USA); the drugs were used as received. The apoferritin from equine spleen (APO) was purchased from Sigma-Aldrich Co. (St Louis, MO, USA). *N*-(3-dimethylaminopropyl)-*N′*-ethylcarbodiimide hydrochloride (EDC), *N*-hydroxysuccinimide (NHS), folic acid, and other chemicals were obtained from Aldrich Chemical Co. (Milwaukee, Wyoming, U.S.A) and were of analytical grade and used as received. Phosphate-buffered saline (PBS) pH 7.4 consisted of 1.8 mM Na_2HPO_4, 137 mM NaCl, 2.7 mM KCl and 10 mM KH_2PO_4. McIlvain buffer (MI buffer) solutions (pH 3.6, 4.6 and 5.6) were prepared from 0.2 M Na_2HPO_4 and 0.1 M citric acid stock solutions. All aqueous solutions were prepared with deionized water (resistivity of 18.2 MΩ cm) purified with a Milli-Q reagent grade water system (Merck Millipore, Billerica, MA, USA).

Apparatus

The fluorescence spectra were recorded using LS55 Spectrometer (Perkin Elmer, Waltham, MA, U.S.A.) equipped with 20 kW Xenon light source operating in 8 μs pulsing mode. Separate monochromators for the incident and detector beams enabled to use monochromatic radiation with wavelengths from 200 to 700 nm. The dual detector system consisted of a photomultiplier tube (PMT) and an avalanche photodiode. The UV–Vis spectra were recorded using a Varian Cary 50 Bio UV–Visible Spectrophotometer (Agilent Technologies, Santa Clara, CA, USA) in the range from 200 nm to 700 nm at room temperature. FTIR spectra of APO nanocages were obtained using Model Nicolet iS-10 FTIR instrument (Thermo Fisher Sci., USA) working in specular mode enabling the reflection from the sensor surface to be analyzed. The circular dichroism (CD) spectra were recorded on a J-715 spectropolarimeter (JASCO, Tokyo, Japan). Far-UV (190–240 nm) recordings were performed in a 0.5 cm pathlength quartz cuvette.

Incorporation of anthracycline drugs to apoferritin nanocages

The four anthracyclines studied have a similar structure and their main pK_a values are close to 8.46 and 7.34, due to the amine group protonation and phenolic dissociation, respectively. Hence, the net charges carried by these anthracycline molecules do not differ significantly. However, the small structural differences, including additional $-O-CH_3$ group at C4, as well as $-CH_2OH$ or $-CH_3$ group at C13, result in considerable differences in drugs lipophilicity and supramolecular interactions with apoferritin protein, thus influencing the encapsulation efficiency. The measurements performed in this work were designed to evaluate the efficiency of encapsulation of anthracyclines under study in APO nanocages.

The encapsulation of anthracycline drugs in APO nanocages was carried out by the disassembly/reassembly protocols based on those developed recently [1-4] and with changes adopted for the drugs used in this study. Briefly, a 5 or 25 µL of 100 mg/mL horse spleen APO (final concentration of APO 1 or 5 mg/mL) was mixed with 10 or 50 µL of 10 mM anthracycline derivative (DOX, EPI, DAU and IDA) in a molar ratio of APO:drug of 1:100. The total volume of the mixtures was 500 µL which was obtained by adding appropriate amounts of Milli-Q water. A 4 µL aliquot of 1 M hydrochloric acid was added to decrease the solution pH to 2.0 and dissociate the APO. Then the pH value was maintained for 15 minutes. Afterward, the pH was slowly increased up to 7.4 using 4 µL of 1 M sodium hydroxide. The resulting solution was stirred under room temperature for 2 hours to encapsulate the anthracycline in the APO. Then, the mixture was rinsed five times with Milli-Q water using Amicon Ultra-0.5 mL 30K (Merck Millipore, Billerica, MA, USA) to remove unbound drug molecules. The obtained anthracycline-containing APO nanocages (APO(drug)) were stored at 4 °C.

Modification of APO(drug) nanocages with folic acid

To improve the effectiveness of APO(drug) nanocages in targeted drug delivery, we have applied a covalent binding of folic acid (FA) to an APO(drug) nanocage using a modification of the standard EDC/NHS reaction. 10 mM FA was dissolved in 5 % $NaHCO_3$. To activate carboxyl groups of the folic acid, 10 µL of FA solution was added to 900 µL of EDC/NHS mixture containing 7 mM EDC and 7

mM NHS in water and the resulting solution was allowed to react at room temperature for 30 minutes. The activated mixture was added to the APO(drug) nanocages, prepared earlier by centrifugation using Amicon Ultra-0.5 mL 3K to remove water, and were incubated overnight at room temperature. The APO(drug)@FA nanocarriers were purified through the Amicon Ultra-0.5 mL 30K and washed 3-times with Milli-Q water. The obtained product was dispersed in water for further characterization and application.

Encapsulation ratio drug:APO

The quantification of encapsulated anthracyclines was performed by determining the concentration of free drugs in solution, separated from APO(drug) NPs by filtration and the initial drug concentration in the encapsulation solution. The concentrations of free drugs were evaluated using fluorescence emission measurements, for λ_{ex} = 480 nm, with calibration data detailed in Figure S3. All the data are expressed as the average of at least three determinations.

Releasing of anthracyclines form APO(drug) nanocages

The kinetics of drug release from APO(drug) was evaluated using PBS buffer pH 7.4 and McIlvain buffers with pH 3.6, 4.6 and 5.6. In brief, 300 µL of the appropriate buffer was added to the APO(drug) and samples were incubated for 6 h at 37 °C. At the pre-set time points (1 h apart), the solution with unbound drug was separated by filtration using Amicon-Ultra-0.5 mL 3K at RT. The container with APO(drug) nanocarriers was replenished with an equal volume of blank buffer (300 µL). The release study was carried out in triplicate for each pH.

Cell culture

Cells were cultured in a humidified incubator at 37 °C with 5% CO_2. The cell lines used in this study: standard HeLa cancer cells, MDA-MB-231 human mammary gland breast cancer adenocarcinoma cells, and MCF10A human mammary non-tumorigenic epithelial cells, were obtained from American Type Culture Collection (Manassas, VA, USA). HeLa and MDA-MB-231 cells were cultured as monolayers

in Dulbecco's Modified Eagle Medium (DMEM) supplemented with 10% fetal bovine serum (FBS). The growth medium for MCF10A cells contained DMEM, horse serum (5 %), epidermal growth factor (EGF, 20 ng/mL), hydrocortisone (0.5 mg/mL), cholera toxin (100 ng/mL), insulin (10 μg/mL). Cells were routinely passed at 80–90 % confluence, using trypsin/EDTA.

Cell death assay

To determine the fraction of dead and apoptotic cells induced by targeted free anthracyclines (DOX, EPI, IDA) and APO nanocages uploaded with encapsulated anthracyclines (DOX, EPI, or IDA), the Annexin V method was used. MDA-MB-231 cells were exposed to the indicated concentrations of anticancer drugs and APO nanocages for 72 h. Media and cells were then collected, pelleted, and processed according to the Muse™ Cell Analyzer Annexin V and Dead Cell Kit instructions (MCH100105). Briefly, 100 µL of cells in suspension were added to tubes. Next, 100 µL of the Muse™ Annexin V & Dead Cell Reagent was added to each tube. Samples were stained for 20 minutes at room temperature in the dark. Approximately 5,000 cells were gated for analysis per sample; triplicate biological replicates were analyzed.

Colony formation assay by crystal violet staining

Colony formation assay is an *in vitro* cell survival assay based on the ability of a single cell to grow into a colony. HeLa, MDA-MB-231, and MCF10A cells were seeded onto six-well plates at a concentration of 150,000 cells/well such that they were ~40% confluent at 24 hours. The cells were then treated with IDA and APO(IDA) at concentrations: 0.0, 0.5 and 1.0 µM of the drug. After the treatment, cells were incubated in 5 % CO_2 atmosphere at 37 °C for 72 h to allow for colony formation. After the 72-hour treatment, pictures of each well on the six-well plates were taken. The cells were then fixed in 3.7 % formaldehyde for 15 minutes. The fixed cells were rinsed with PBS and stained for 10 minutes using 0.1 % crystal violet. After staining, the plates were rinsed with water to remove the staining solution and allowed to air dry. To quantify, the cells were solubilized in 1 % SDS and the absorbance at 590 nm was measured. The percentage of cell survival (PCS) was calculated as follows:

$$PCS = \frac{No.\ of\ cells\ after\ treatment}{No.\ of\ cells\ in\ UC} \times 100 \quad (1)$$

where UC stands for the untreated control.

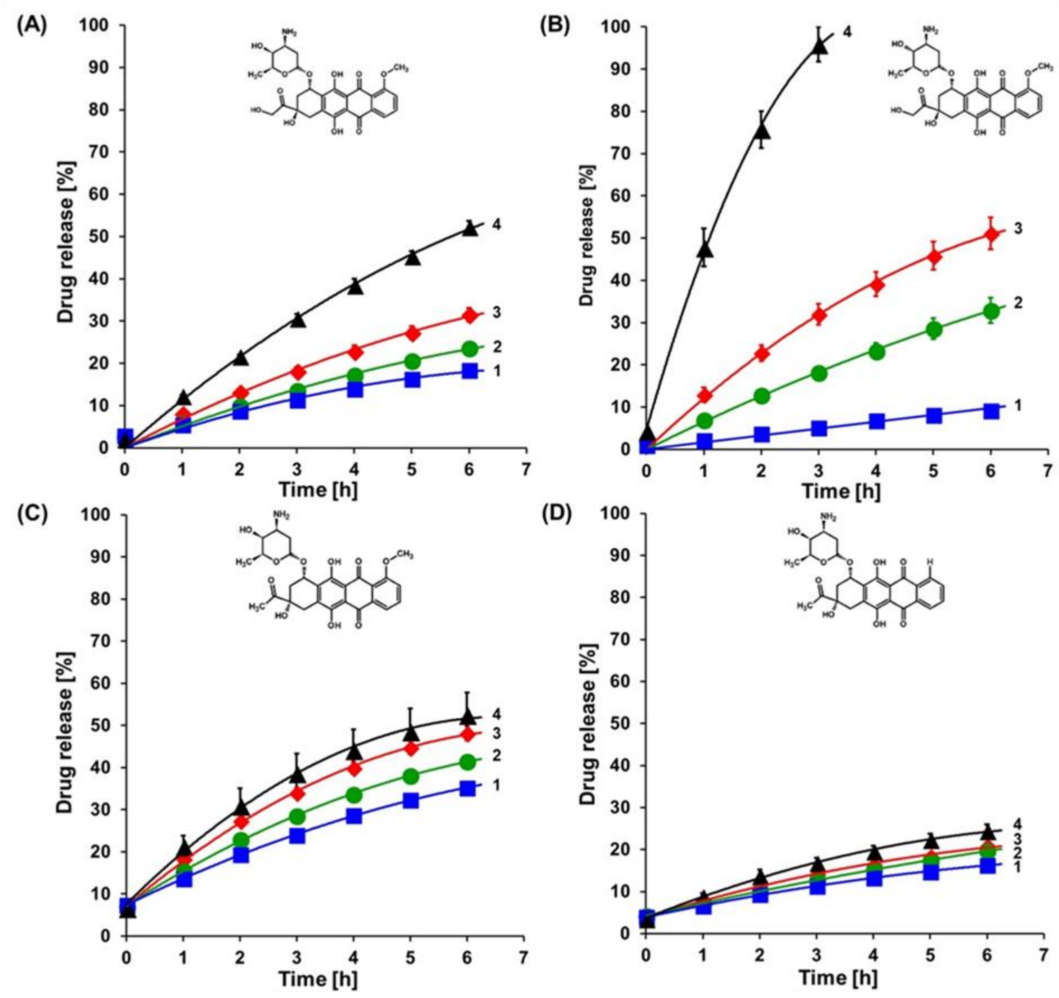

Figure S1. Temporal evolution of (**A**) DOX, (**B**) EPI, (**C**) DAU and (**D**) IDA release form 1 mg/mL APO nanocages in solution of pH (**1**, ■) 7.4, (**2**, ●) 5.6, (**3**, ♦) 4.6 and (**4**, ▲) 3.6. The release function *R* was determined from the fluorescence emission spectra of the released drug from the APO cavity (excitation at 480 nm, emission at 570 nm). Values are expressed as the mean ± standard deviation (n = 5), 0.1 < SD < 5.5.

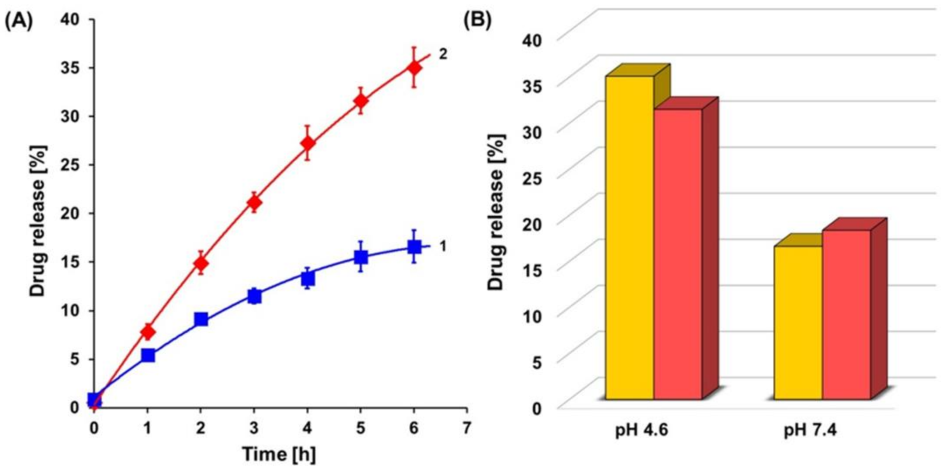

Figure S2. (**A**) Temporal evolution of DOX release from the APO(DOX)@FA nanocages in solution of pH 7.4 (**1**, ■) and 4.6 (**2**, ♦). (**B**) Comparison of DOX release at pH 4.6 and 7.4 after 6 h from the APO(DOX) bare (red column) and modified with FA (yellow column) nanocages. The release function R was determined from the fluorescence emission spectra of the released drug from the APO cavity (excitation at 480 nm, emission at 570 nm). Values expressed as the means ± standard deviation (n = 3), 0.1 < SD < 2.0.

References

1. Dostalova S, Cerna T, Hynek D, Koudelkova Z, Vaculovic T, Kopel P, et al., Site-direct conjugation of antibodies to apoferritin nanocarrier for targeted drug delivery to prostate cancer cells. *ACS Appl Mater Interfaces*. 2016;**8**:14430-14441.

2. Dostalova S, Vasickova K, Hynek D, Krizkova S, Richtera L, Vaculovicova M, et al., Apoferritin as ubiquitous nanocarrier with excellent shelf life. *Int J Nanomed*. 2017;**12**:2265-2278.

3. Ruozi B, Veratti P, Vandelli MA, Tombesi A, Tonelli M, Forni F, et al., Apoferritin nanocage as streptomycin drug reservoir: technological optimization of a new drug delivery system. *Int J Pahramc*. 2017;**518**:281-288.

4. Falvo E, Tremante E, Fraioli R, Leonetti C, Zamparelli C, Boffi A, et al., Anti-body conjugates: targeting melanoma with cisplatin encapsulated in protein-cage nanoparticles based on human ferritin. *Nanoscale* 2013;**5**:12278-12285.
